# Algor‐Ethics in Diabetes Care: Mapping the Route

**DOI:** 10.1002/dmrr.70139

**Published:** 2026-02-18

**Authors:** Joshua Bemporad, Francesco De Domenico, Paolo Pozzilli

**Affiliations:** ^1^ Campus Bio‐Medico University Rome Italy; ^2^ Fondazione Policlinico Universitario Campus Bio‐Medico Rome Italy; ^3^ Blizard Institute Centre of Immunobiology Barts and the London School of Medicine Queen Mary University of London London UK

**Keywords:** Algor‐ethics, algorethics, artificial intelligence, diabetes, diabetes care, ethics, machine learning

## Abstract

Diabetes mellitus represents a multifaceted global health challenge, frequently coexisting with obesity, cardiovascular complications, and metabolic disorders. Effective management requires individualised, evidence‐based decisions informed by an array of clinical, genetic, and lifestyle data. With the rapid growth of digital health technologies, artificial intelligence (AI) and algorithmic systems have emerged as powerful tools to support clinicians in diagnosis, treatment planning, and risk stratification. While AI shows promise in improving diabetes outcomes and health system efficiency, its integration into patient care is not without ethical and epistemic challenges. Algorithmic decision‐making can influence therapeutic strategies, sometimes without full transparency or adequate oversight, potentially compromising human values such as autonomy, justice, and trust. In this context, the discipline of ‘Algor‐ethics’, a term coined to describe the intersection of algorithmic systems and ethical principles, becomes critical. This article explores the foundational concepts of Algor‐ethics applied to diabetes care, analyzes the current state of AI integration, and highlights the epistemic and ethical implications of algorithmic decision‐making. Emphasis is placed on developing a framework that ensures AI is implemented safely, equitably, and responsibly, particularly for complex patients with diabetes.

## 1 Introduction

In recent years, artificial intelligence (AI), especially machine learning (ML) and deep learning (DL), has transformed how clinical data is processed, offering clinicians unprecedented tools for pattern recognition, predictive analytics, and real‐time decision support. In the context of diabetes care, machine learning–based systems have been increasingly applied to glucose forecasting from continuous glucose monitoring data, insulin dosing support, automated retinal screening, and patient phenotypic risk stratification, demonstrating promising performance across multiple clinical settings [1, 2]. Diabetes represents a paradigmatic condition for the ethical challenges posed by algorithmic decision‐making due to its chronicity, data intensity, and reliance on patient‐facing technologies. Clinical decision support systems (CDSS), powered by AI, provide personalised recommendations based on patient data and guideline‐informed recommendations, with growing evidence that such systems can support clinicians in complex therapeutic decisions without replacing clinical responsibility [3]. When integrated into electronic health records (EHRs), these tools can enhance clinical workflows, reduce errors, and optimise therapeutic strategies. The clinical value of these tools, however, is contingent upon their reliability, transparency, and appropriate integration into human decision‐making, underscoring the need for a balanced interaction between clinician expertise and algorithmic support [4]. Despite technological advances, several ethical tensions persist. AI systems often operate as ‘black boxes’, lacking transparency in their decision‐making pathways [5]. They may also perpetuate biases embedded in training data, leading to unequal treatment outcomes, especially among underrepresented populations [6]. Moreover, the potential for automation bias, where clinicians defer too readily to algorithmic outputs, raises concerns about erosion of clinical judgement [7]. To address these challenges, the emerging field of ‘Algor‐ethics’ seeks to incorporate ethical norms into the design, development, and deployment of AI [8]. The term ‘algor‐ethics’ (or ‘algorethics’) was coined by the Italian Franciscan theologian and ethicist Paolo Benanti around 2018 [9]. Benanti argues that algorithms shape decisions in healthcare, politics, the economy, and daily life, so ethics must be integrated in their design, training, deployment, and oversight (‘from the ethics of technology to algor‐ethics’). As it is described in the article by Montomoli et al. with regard to critical care [8], we believe that diabetes care requires a structured ethical framework that keeps clinicians in control, mitigates bias, and embeds AI within human‐centred governance structures.

This article is conceived as a conceptual and ethical analysis rather than a technical or systematic review of artificial intelligence methods. Its primary aim is to explore how algorithmic decision‐making intersects with epistemic, ethical, and clinical dimensions that are particularly salient in diabetes care. Rather than cataloguing technologies, the manuscript focuses on ‘Algor‐ethics’ as a framework to guide responsible governance, accountability, and clinician–patient interaction in an era of increasingly algorithm‐mediated diabetes management.

## 2 Domains of AI in Diabetes Care

AI applications in diabetes care span multiple distinct domains, each associated with specific ethical and epistemic challenges. In this section, we will illustrate relevant examples to advance our narrative.

### 2.1 AI and Insulin Titration

Artificial intelligence plays an emerging role in insulin titration by providing clinical decision support systems that deliver personalised, real‐time recommendations for insulin dose adjustments, aiming to optimise glycaemic control and reduce therapeutic inertia. AI‐driven titration tools have demonstrated noninferiority to expert endocrinologists in randomised clinical trials for hospitalised patients with type 2 diabetes, with comparable time‐in‐range (TIR) and safety profiles, and high physician satisfaction regarding usability and effectiveness. These systems leverage patient‐specific data, including glucose measurements, insulin regimens, and comorbidities, to dynamically adjust basal‐, basal‐bolus‐, and premixed insulin regimens, and are adaptable to both insulin‐naive and insulin‐experienced populations [10].

In outpatient settings, AI‐based titration platforms such as d‐Nav have shown superior glycaemic control compared to standard care, automating dose adjustments based on glucose patterns and reducing the need for frequent physician intervention [11]. Reinforcement learning frameworks and other machine learning approaches have further improved accuracy in insulin dose optimisation, outperforming traditional formula‐based methods [12].

For type 1 diabetes, AI‐powered decision support systems for pump and multiple daily injection therapy have been validated in multicenter trials, demonstrating noninferiority to physician‐guided titration in both efficacy and safety, and supporting their use in routine clinical practice [13]. AI‐assisted titration also addresses barriers such as clinical inertia and the complexity of individualised dosing, and can be integrated into primary care workflows to support non‐specialist providers.

### 2.2 AI and CGM

Widely used CGM (continuous glucose monitoring) systems, primarily offer real‐time glucose readings, trend arrows, and customisable alerts for impending hypo or hyperglycemia, but do not natively include advanced predictive modelling for future glucose values or hypoglycemia risk in their standard consumer offerings. Instead, these systems rely on trend analysis and alarms based on current and recent glucose data [14]. Predictive models for glucose forecasting and hypoglycemia prevention are now included in some CGM sensors that are commercially available to the public. These systems provide real‐time glucose predictions, including two‐hour glucose forecasts and specific hypoglycemia prediction features, using artificial intelligence directly within the CGM ecosystem [15].

### 2.3 AI and Diabetes Complications

AI plays a rapidly expanding role in the diagnosis and management of diabetic retinopathy (DR), primarily by enabling automated screening, improving diagnostic accuracy, and supporting risk stratification. Deep learning algorithms trained on large retinal image datasets can autonomously detect referable DR with sensitivity and specificity comparable to human graders, often exceeding 85% for both metrics in prospective studies and meta‐analyses [16]. AI‐based systems are now FDA‐approved for autonomous diabetic retinopathy screening in primary care settings in the US and have been implemented in several countries, facilitating earlier detection and improved screening coverage, especially in resource‐limited or high‐volume settings [17].

Artificial intelligence (AI) plays an emerging role in diabetic nephropathy (diabetic kidney disease, DKD) by enabling earlier detection, risk stratification, prediction of progression, and streamlining histopathological assessment. AI models utilising electronic health records, clinical metadata, and retinal imaging have demonstrated moderate to high accuracy (AUROC 0.701–0.914) in predicting DKD onset and progression, outperforming traditional risk scores in some studies [18]. Notably, deep learning applied to retinal images, leveraging the correlation between diabetic retinopathy and DKD, offers a resource‐efficient approach for opportunistic DKD screening during routine diabetic retinopathy assessments [19].

Similarly, machine learning and deep learning algorithms, have demonstrated the ability to analyse multimodal data, including corneal confocal microscopy images and electronic health records, to detect and predict diabetic peripheral neuropathy (DPN) with promising accuracy [20].

### 2.4 AI and CDSS

AI‐driven clinical decision support systems (CDSS) enable data‐driven, personalised diabetes management by synthesising patient data from electronic health records, continuous glucose monitoring, and wearable devices to support diagnosis, risk stratification, treatment selection, and complication prediction. These systems facilitate individualised therapy optimisation, such as recommending medication adjustments based on predicted glycaemic response, thereby minimising therapeutic inertia and improving clinical outcomes [21]. An example of AI‐driven CDSS implemented in diabetes care, is a five‐drug class model that uses routinely available clinical features to optimise prescribing for type 2 diabetes. The model predicts the relative glycaemic effectiveness of the following five major non‐insulin drug classes: dipeptidyl peptidase‐4 inhibitors, glucagon‐like peptide‐1 receptor agonists, sodium–glucose co‐transporter‐2 inhibitors, sulfonylureas, and thiazolidinediones [22].

### 2.5 AI and Risk of Developing Diabetes

Artificial intelligence is used to predict the risk of developing diabetes by applying machine learning algorithms to large, multidimensional datasets that include clinical, demographic, genetic, behavioural, and imaging data. These models, such as decision trees, neural networks, support vector machines, and ensemble methods such as XGBoost and Extra Trees, analyse patterns and interactions among established risk factors (e.g., age, BMI, glucose levels, family history, blood pressure, lipid profiles, and lifestyle behaviours) to estimate an individual's future risk of diabetes [23].

### 2.6 AI in Scientific Publications

A survey of 59 Editors‐in‐Chief found that about half of medical journals already use AI, mainly for plagiarism detection and fact checking, with time and cost savings seen as key advantages. At the same time, editors are worried about bias, accountability, and overreliance on AI, though most expect AI to play a major role in medical publishing within the next decade [24].

Each of these domains differs in acceptable uncertainty, explainability requirements, and clinical risk, underscoring the need for domain‐specific ethical evaluation rather than a one‐size‐fits‐all approach.

## 3 Epistemic and Ethical Challenges of Algorithmic Decision‐Making

### 3.1 Epistemic Uncertainty and Explainability

AI‐driven systems, particularly those based on deep learning, often lack explainability, the ability for users to understand the rationale behind outputs [5]. As previously described, in diabetes care, AI‐driven systems are increasingly used to interpret continuous glucose monitoring data, predict glycaemic excursions, support insulin dosing decisions, and estimate the risk of long‐term complications. These models often integrate heterogeneous data streams, including glucose time series, lifestyle factors, comorbidities, and treatment history, making it difficult for clinicians to fully reconstruct the rationale underlying a specific recommendation. This lack of explainability complicates clinical validation, especially in patients with multimorbidity or atypical disease trajectories, and may lead to either over‐reliance on or inappropriate rejection of algorithmic outputs. Furthermore, the probabilistic nature of many AI models introduces a degree of uncertainty that is difficult to reconcile with traditional evidence‐based paradigms. Without a clear understanding of an algorithm's limitations, clinicians risk either over‐reliance or under‐utilisation of these tools [25].

### 3.2 Ethical Concerns: Autonomy, Consent, and Trust

AI in healthcare challenges established ethical norms by shifting how decisions are made and who is accountable for them [26]. Respect for patient autonomy demands that individuals be involved in decisions about their care. However, if AI recommendations are opaque or presented as definitive, patients may feel excluded or coerced, undermining their informed consent. Trust is a cornerstone of clinical care. If algorithms are seen as unpredictable or biased, they may erode patient trust in healthcare providers or institutions. These ethical tensions are amplified in chronic diseases such as diabetes, where patients interact with digital tools and algorithmic recommendations on a daily basis rather than during isolated clinical encounters. Long‐term reliance on AI‐mediated decisions may reshape patient autonomy, redistribute responsibility between patients and clinicians, and alter the clinician–patient relationship. Ensuring that patients remain informed participants in decision‐making processes is therefore essential for preserving trust and meaningful consent in AI‐supported diabetes care.

### 3.3 Bias, Fairness, and Representativeness

AI systems trained on unrepresentative datasets may systematically disadvantage certain groups, such as ethnic minorities or patients with atypical disease trajectories [27]. Bias and lack of representativeness are particularly concerning in diabetes care, given the marked heterogeneity of the disease and the well‐documented underrepresentation of ethnic minorities, older adults, socioeconomically disadvantaged populations, and patients with atypical phenotypes in many datasets. Algorithmic models trained on such data may systematically misestimate risk, misclassify disease subtypes, or generate suboptimal therapeutic recommendations, thereby reinforcing existing health disparities. Addressing fairness in diabetes‐related AI therefore requires deliberate efforts towards inclusive data collection, bias auditing, and continuous model recalibration.

### 3.4 Automation Bias and the Role of Clinicians

While AI is intended to augment clinical reasoning, there is growing concern about automation bias, the tendency of users to favour algorithmic outputs over their own judgement [7]. This can lead to diagnostic or therapeutic errors, particularly when clinicians defer to AI without critical evaluation [28]. A human‐in‐the‐loop approach, where clinicians remain actively involved in interpreting and validating AI outputs, is essential. It not only preserves the integrity of medical decision‐making but also ensures that ethical norms such as beneficence and non‐maleficence, are upheld [29]. In diabetes management, automation bias may manifest when clinicians defer algorithmicing insulin dosing suggestions, risk alerts, or treatment recommendations without sufficient critical appraisal. Given the potential consequences of inappropriate dosing or delayed intervention, maintaining an active human‐in‐the‐loop approach is essential to ensure that AI augments rather than supplants clinical judgement.

## 4 Algor‐Ethics Principles Applied to Diabetes Management

### 4.1 Human‐in‐the‐Loop Principle

The ethical integration of AI in diabetes management fundamentally relies on the human‐in‐the‐loop principle, whereby algorithms support ‐ but do not replace ‐ clinical reasoning. While AI systems can efficiently analyse large datasets for tasks such as retinopathy screening, nephropathy risk prediction, or glycaemic pattern recognition, they remain vulnerable to data limitations, contextual blind spots, and model bias. Clinician oversight is therefore essential to interpret outputs, validate recommendations, and adapt decisions to individual patient contexts. For example, AI‐based retinal image analysis systems can achieve high sensitivity for diabetic retinopathy detection, but false positives and negatives remain, necessitating clinician validation before clinical action is taken [16]. Similarly, risk stratification algorithms for complications must be interpreted in the context of individual patient histories, comorbidities, and social determinants, which AI may not fully capture. Clinical decision support systems powered by AI integrate data from continuous glucose monitoring, insulin pumps, wearables, electronic health records, and lifestyle factors to recommend insulin adjustments and flag glycaemic risks. However, these systems are designed to support, not replace, clinician decision‐making, and clinicians can override AI recommendations as needed. Maintaining human oversight mitigate risks of automation bias, where clinicians might over‐rely on AI outputs, thus upholding ethical principles such as beneficence and non‐maleficence.

### 4.2 Algorithmic Stewardship

Algorithmic stewardship involves governance frameworks overseeing AI development, deployment, and monitoring [30]. In diabetes care, stewardship ensures that AI tools remain safe, effective, and equitable throughout their lifecycle. For example, AI algorithms used for diabetic retinopathy screening must be regularly audited to maintain accuracy across diverse populations. Multidisciplinary teams, including endocrinologists, ophthalmologists, data scientists, and ethicists, review algorithm performance, update models with new data, and audit for biases [16]. In resource‐limited settings, stewardship frameworks will guide responsible AI deployment to ensure equitable access and effectiveness. Regular audits can mitigate risks such as algorithmic drift, where model performance degrades due to changes in patient demographics or clinical practices. In diabetes care, algorithmic stewardship is particularly relevant given the dynamic nature of clinical data and treatment strategies. Continuous auditing is required to ensure that AI tools maintain performance across diverse patient populations and evolving standards of care.

### 4.3 Traceability and Transparency

Traceability and transparency require clear documentation and explainability of AI algorithms, enabling clinicians and patients to understand how recommendations are generated. Explainable AI (xAI) techniques applied to diabetes risk prediction models highlight which factors influence risk scores, allowing clinicians to validate AI suggestions. Transparency about limitations, such as reduced accuracy in paediatric patients or those with atypical diabetes phenotypes, enables clinicians to avoid over‐reliance and engage patients in informed discussions. This is supported by recent systematic reviews and position statements in the medical literature, which emphasise that xAI methods such as SHAP, LIME, and Grad‐CAM are increasingly used to improve the transparency and clinical interpretability of AI models in diabetes care. These approaches allow clinicians to understand which features most influence a model's prediction, supporting validation and shared decision‐making [23].

### 4.4 Customisation and Patient‐Centeredness

Diabetes is heterogeneous, with variable phenotypes and treatment responses. Customisation refers to the ability of AI systems to adapt recommendations beyond rigid guideline categories by integrating individual patient characteristics, treatment responses, and contextual factors. In diabetes care, this may include adjusting insulin dosing strategies, monitoring intensity, or therapeutic sequencing in patients whose clinical trajectories do not align with standard type 1 or type 2 diabetes algorithms. While such tailoring can enhance patient‐centred care, it also requires transparency and clinician oversight to ensure that individualised recommendations remain evidence‐based and ethically sound. These systems leverage machine learning algorithms to identify patterns in glycaemic variability, anticipate hypoglycaemic or hyperglycaemic events, and optimise therapy in real time, supporting both patient self‐management and clinician‐led care [31]. Customisation supports shared decision‐making, respect autonomy, and addresses disparities by adapting care to diverse populations.

### 4.5 Accountability

Accountability ensures clear responsibility for AI‐driven clinical decisions among providers, institutions, and developers. For example, when AI flags a high risk of diabetic nephropathy, clinicians must appraise alerts, confirm diagnoses, and decide interventions. Organisations implement reporting systems for AI‐related errors, enabling quality improvement. Regulatory frameworks require AI systems to meet safety, efficacy, and surveillance standards. Clinicians retain ultimate responsibility, while developers maintain algorithm quality and transparency, fostering safety and trust. Accountability in AI‐supported diabetes care requires a clear delineation of responsibility among clinicians, healthcare institutions, and developers. While algorithms may generate recommendations or alerts, clinicians retain ultimate responsibility for clinical decisions. At the same time, developers and institutions must ensure algorithmic quality, transparency, and appropriate monitoring to support safe and ethical use. Table 1 summarises the core algor‐ethics principles and their practical implications for diabetes care.

**TABLE 1:** Application of algor‐ethics principles.

Section	Key points
Human‐in‐the‐loop	AI should support clinical reasoning rather than replace it; AI outputs must always be interpretable.
Algorithmic stewardship	Continuous auditing is necessary to ensure equitable application across diverse patient demographics.
Traceability & transparency	AI systems must document which rules were applied, how they were adapted, and where limitations lie.
Customisation	AI should personalise care while staying grounded in evidence‐based recommendations.
Accountability	Clear lines of responsibility must be defined when AI‐influenced decisions result in harm.

## 5 Regulatory and Policy Implications

### 5.1 Overview of Existing Regulations and Guidelines

The rapid integration of artificial intelligence into healthcare has prompted the development of comprehensive regulatory frameworks to ensure safety, efficacy, and ethical standards. Key regulations and guidelines include:

- European Union Artificial Intelligence Act (2024): The EU AI Act is the first comprehensive legal framework specifically addressing AI, including high‐risk applications in healthcare. It mandates requirements for transparency, risk management, human oversight, and post‐market monitoring for AI systems used in clinical settings. The Act categorises AI systems by risk level and imposes strict obligations on high‐risk AI, such as clinical decision support tools used in diabetes care. It also complements existing data protection laws such as the General Data Protection Regulation (GDPR), ensuring patient data privacy and security [32].
- FDA Guidelines (United States): The U.S. Food and Drug Administration (FDA) regulates AI‐based medical devices under the Software as a Medical Device (SaMD) framework. The FDA emphasises safety, effectiveness, and continuous monitoring, with recent proposals considering the regulation of autonomous AI systems in clinical practice [33].
- Global and National Approaches: Many countries are developing or updating their own AI strategies and regulations, focussing on data security, privacy, algorithmic transparency, and accountability. However, regulatory maturity varies, and gaps remain, especially regarding adaptive and autonomous AI systems [34].

These frameworks generally require that AI systems in healthcare be explainable, robust, and subject to human oversight, with clear mechanisms for reporting adverse events and updating algorithms as new evidence emerges.

### 5.2 Role of Oversight Bodies

Oversight bodies are gradually emerging to support the implementation of ethical and regulatory standards in healthcare. While still in development, they are gaining importance in response to evolving policies such as the EU Artificial Intelligence Act (2024) [35]:

- Governance and Stewardship: These entities will be responsible for overseeing the entire lifecycle of AI systems, from selection and validation to deployment and monitoring. They will ensure that AI tools will align with clinical guidelines, institutional policies, and regulatory requirements.
- Bias Auditing and Equity Monitoring: Oversight bodies will conduct regular audits to detect and address biases, ensuring equitable care across diverse patient populations, including those with diabetes from minority or underserved groups.
- Education and Training: These entities will provide ongoing education for healthcare professionals, equipping them to interpret AI outputs, recognise limitations, and maintain human oversight in decision‐making.
- Incident Reporting and Quality Improvement: These bodies will establish protocols for reporting AI‐related errors or adverse events, facilitate continuous quality improvement and foster a culture of safety and accountability.

By integrating these regulatory and policy measures, healthcare systems can harness the benefits of AI in diabetes care while safeguarding patient rights, promoting equity, and maintaining public trust. In the context of diabetes care, while there are no diabetes‐specific AI regulations or guidelines explicitly cited yet, the integration of AI into healthcare, including diabetes management, is increasingly governed under broader regulatory frameworks, particularly within the European Union.

### 5.3 Privacy and Cybersecurity

AI‐driven medical devices for diabetes care are vulnerable to cybersecurity threats including unauthorised access, data breaches, manipulation of device functions and exposure of sensitive patient data, exacerbated by wireless connectivity and cloud integration that expand the attack surface and threaten confidentiality, integrity and availability. Specific weaknesses encompass poor credential management, hard‐coded credentials, unpatched software, insecure communication channels and AI‐related risks such as adversarial attacks, model manipulation and data poisoning. All these factors can degrade clinical decision accuracy or introduce biases, potentially endangering lives, especially when devices are used for insulin delivery or titration. Fortunately, regulatory bodies such as the FDA have established governance, risk and compliance standards to address these risks, emphasising the need for continuous risk assessment, secure software development, and explainable AI frameworks to enhance transparency and trust. The Diabetes Technology Society highlights the importance of rigorous cybersecurity standards, such as the IEEE 2621, and recommends countermeasures including security‐by‐design with robust encryption and memory protections, explainable AI for transparency, continuous risk assessments and vulnerability management via platforms like Malware Information Sharing Platform (MISP), machine learning‐based intrusion detection, granular user data controls with end‐to‐end encryption and ongoing testing/monitoring to ensure compliance, patient safety and trust [36, 37].

## 6 Use of Clinical Guidelines in AI‐Driven Diabetes Care

### 6.1 Clinical Algorithms Are Already Algorithmic Decision Systems

Both the American Diabetes Association (ADA) and the European Association for the Study of Diabetes (EASD) offer flowcharts and stepwise algorithms for diagnostic and therapeutic decisions, such as using haemoglobin A1c and fasting glucose levels to diagnose type 2 diabetes and recommending metformin as a first‐line therapy in type 2 diabetes [38]. These structured decision trees are often embedded in Clinical Decision Support Systems (CDSS).

### 6.2 AI Can Scale and Amplify These Algorithms: For Better or Worse

AI models have the capacity to enhance the application of these guidelines by integrating real‐time data (e.g., from continuous glucose monitors or wearables), identifying patterns not discernible by humans, and tailoring recommendations for subpopulations not explicitly addressed by existing guidelines. However, these benefits are accompanied by risks, such as amplifying biases embedded in original guidelines, reducing clinical transparency, and potentially undermining patient autonomy through over‐reliance on AI‐driven recommendations.

### 6.3 No Current Regulatory Oversight of These Guidelines When Integrated in AI Tools

The American Diabetes Association (ADA) and the European Association for the Study of Diabetes (EASD) have not published formal position statements or clinical guidelines specifically dedicated to artificial intelligence (AI) in diabetes care as of December 2025. However, both organisations have acknowledged the growing role and potential of AI in diabetes management through consensus reports, expert reviews, and collaborative initiatives.

Recent consensus from the European Diabetes Forum (EUDF), which works closely with the EASD, highlights that AI‐driven clinical decision support systems (AI‐CDSS) are expected to deliver significant benefits in diabetes care, including minimising treatment inertia and optimising clinical outcomes. The EUDF working group emphasises the need for robust regulatory frameworks and careful integration of AI technologies to ensure safety, efficacy, and ethical use in clinical practice. They have issued recommendations to guide the safe and effective implementation of AI‐CDSS, focussing on personalised medicine, improved outcomes, and support for primary care providers [21].

This regulatory gap introduces several ethical questions: Who is accountable if harm occurs through an app using these guidelines? How can fairness be maintained when guidelines are applied to populations underrepresented in original trials? Is human oversight adequately preserved in the clinical workflow? Regulatory bodies like the FDA or EMA only evaluate these algorithms when they are embedded in certified medical software, and even then, the focus is often on software functionality rather than clinical content. We believe that a critical role must be played by the diabetes associations such as ADA and EASD in collaboration with the international regulatory bodies in the design of specific guidelines, in order to provide guidance and clarity to clinicians as well as patients with diabetes. Key future research and implementation priorities are summarised in Table 2.

**TABLE 2:** Future directions and research needs.

Section	Key points
Diverse data collection	Proactively gather data from underrepresented populations. Collaborate with global and community health organisations to access broader datasets.
Bias detection and mitigation	Develop tools to identify and quantify dataset/model bias. Research fairness interventions: Reweighting, data augmentation, adversarial debiasing.
Standardisation and transparency	Establish standards for data documentation (e.g., “datasheets for datasets”). Encourage open sharing of de‐identified datasets. Implement systems for ongoing model evaluation to detect emerging biases, especially in evolving diabetes care.
Human‐centred design	Involve clinicians and patients in the design/testing of explanation interfaces. Diabetes society’s involvement. Investigate effects of different explanation formats (visual, textual, interactive) on understanding and trust.
Communication training	Develop resources/training for clinicians on communicating AI recommendations and uncertainties. Create patient‐friendly guidelines to explain algorithmic limitations and risks. Diabetes associations must play a pivotal role.
Regulatory and ethical frameworks	Advocate for regulations ensuring a minimum level of explainability/transparency for AI tools. Explore ethical implications of partial explainability and the right to an explanation.

## 7 Conclusions

Algor‐ethics will play a pivotal role in shaping the future of healthcare in diabetes. Addressing bias through better data and continuous monitoring is essential for fairness and equity. Enhancing explainability and communication will foster trust, support shared decision‐making, and ensure that AI augments, rather than undermines, clinician‐patient relationships. Ongoing research, interdisciplinary collaboration, and patient‐centred approaches are key to realising the promise of ethical and effective healthcare algorithms.

## Author Contributions

J.B. and F.D.D. conducted the research. J.B., F.D.D. and P.P. contributed to data interpretation. F.D.D., J.B. and P.P. performed the final review of the manuscript. P.P. conceived the manuscript structure, supervised all steps and provided critical revisions. All authors have read and approved the final version of the manuscript.

## Funding

The authors have nothing to report.

## Conflicts of Interest

J.B. declares no conflicts of interests. F.D.D. declares no conflicts of interest. P.P. declares to be a consultant for Dompé Farmaceutici S.p.A.

## Data Availability

Data sharing not applicable to this article as no datasets were generated or analysed during the current study.
